# Root exudates influence rhizosphere fungi and thereby synergistically regulate *Panax ginseng* yield and quality

**DOI:** 10.3389/fmicb.2023.1194224

**Published:** 2023-07-20

**Authors:** Jin Sun, Jing Yang, Shuyue Zhao, Qian Yu, Lili Weng, Chunping Xiao

**Affiliations:** School of Pharmaceutical Sciences, Changchun University of Chinese Medicine, Changchun, China

**Keywords:** *Panax ginseng*, root exudates, rhizome biomass, ginsenosides, fungal community structure

## Abstract

Root exudates contain a complex array of primary and specialized metabolites that play important roles in plant growth due to their stimulatory and inhibitory activities that can select for specific microbes. In this study, we investigated the effects of different root exudate concentrations on the growth of ginseng (*Panax ginseng* C. A. Mey), ginsenoside levels, and soil fungal community composition and diversity. The results showed that low root exudate concentrations in the soil promoted ginseng rhizome biomass and ginsenoside levels (Rg1, Re, Rf, Rg2, Rb1, Ro, Rc, Rb2, Rb3, and Rd) in rhizomes. However, the rhizome biomass and ginsenoside levels gradually decreased with further increases in the root exudate concentration. *ITS* sequencing showed that low root exudate concentrations in the soil hardly altered the rhizosphere fungal community structure. High root exudate concentrations altered the structure, involving microecological imbalance, with reduced abundances of potentially beneficial fungi (such as *Mortierella*) and increased abundances of potentially pathogenic fungi (such as *Fusarium*). Correlation analysis showed that rhizome biomass and ginsenoside levels were significantly positively correlated with the abundances of potentially beneficial fungi, while the opposite was true for potentially pathogenic fungi. Overall, low root exudate concentrations promote the growth and development of ginseng; high root exudate concentrations lead to an imbalance in the rhizosphere fungal community of ginseng and reduce the plant’s adaptability. This may be an important factor in the reduced ginseng yield and quality and soil sickness when ginseng is grown continuously.

## 1. Introduction

Ginseng (*Panax ginseng* C. A. Mey) is mainly found in China, Korea, and Russia, and it is a valuable traditional medicinal herb in East Asia (Abid et al., 2021). With the surge in demand for medicine, the sources of wild ginseng are declining rapidly, and artificial cultivation has become inevitable (Dong et al., 2018). However, this planting method seriously restricts the healthy and sustainable development of the ginseng industry due to soil sickness (Xiao et al., 2016). In short, the high demands on soil quality result in ginseng not being able to grow on the same piece of land for several years in a row (Bao et al., 2022). Ginseng often suffers from soil-borne diseases that damage its root system, eventually leading to a significant reduction in yield and quality (Lei et al., 2017). Therefore, understanding the mechanisms underlying ginseng soil sickness is key in order to improve the yield and quality of ginseng and to break the bottleneck in the development of the ginseng industry.

Root exudates are an important cause of soil sickness (Liu et al., 2020). Chemical substances released from plant root exudates include high- and low-molecular-weight compounds of various chemical classes, such as organic acids, alcohols, sugars, phenols, and hormones (Baetz and Martinoia, 2014). In the soil environment, microbial communities are often influenced by host plant root exudates that not only attract or repel pathogenic bacteria in the swimming stage, but also stimulate or inhibit the germination of non-swimming propagules (Lei et al., 2017). In recent years, the interactions between root exudates and rhizosphere microorganisms and the associated mechanisms have attracted the attention of many researchers (Zhao et al., 2021; Upadhyay et al., 2022). Research has shown that plants use their root exudates to regulate the nearby soil environment, shape the microbiota, and communicate with microorganisms (Salem et al., 2022). Although many aspects of this process remain controversial, a wealth of evidence has indicated the ability of root exudates to regulate plant growth and development by shaping the rhizosphere microbiota (Reinhold-Hurek et al., 2015). For example, Zhou et al. (2023) found that suitable intercropping systems improved the adaptability of intercropped plants (tomato) by using signaling chemicals released by root exudates (potatoonion) to alter recruitment of the rhizosphere microbiota. Luo et al. (2022) showed that when the aboveground part of *Panax notoginseng* was infected by foliar pathogens, it enhanced its secretion of organic acids, sugars, and amino acids in its root exudates, inhibited soil-borne pathogens, enriched beneficial microorganisms, and reduced underground soil-borne diseases.

However, plant root exudates may also have a negative impact on plant development and reduce plant adaptation to pathogens (Yu et al., 2022). An increasing number of studies have shown that ginseng root exudates can cause an imbalance in soil microbial communities, outbreaks of soil-borne diseases, and deterioration of soil physicochemical properties (Li et al., 2020; Tong et al., 2021). For example, saponin-like root exudates cause ginseng root cell death and the release of cellobiose and d-galacturonic acid into the soil, and the accumulation of these compounds increases the number of pathogenic bacteria such as *Ilyonectria* and reduces the number of beneficial microorganisms such as *Pseudomonas* and *Streptomyces* in the soil (Xu et al., 2021). Phenolic acid root exudates are thought to have a greater impact on rhizosphere microorganisms than other secretory compounds such as sugars and amino acids. Phenolic acid can lower soil pH and increase the abundance of phytopathogenic fungi such as *Fusarium*, *Gibberella*, and *Ilyonectria*, causing ginseng root rot (Badri et al., 2013). Root rot is one of the common soil-borne diseases of ginseng, with an incidence of up to 30% in the field, and the incidence increases with age (Bischoff and Goodwin, 2022). Apparently, ginseng root exudates can induce changes in rhizosphere abiotic and biotic properties that in turn affect root growth, in a plant-soil negative feedback mechanism (van der Putten et al., 2013). This feedback plays an important role in maintaining plant diversity and driving community dynamics in natural ecosystems, but it is an important limiting factor for crop productivity in intensive agricultural systems (Crawford et al., 2019; Pizano et al., 2019). Therefore, to ensure the healthy and sustainable development of the ginseng industry, it is necessary to explore the mechanisms underlying the feedback effects of root exudates in plant-soil systems.

Soil microorganisms are the main engines in agroecosystems, driving the turnover of nutrients in the soil (Nie et al., 2018). Fungi include a variety of functional groups (decomposers, mycorrhizal fungi, etc.) and play an important role in soil (Zhao et al., 2023). Although many factors may lead to increased disease stress and decreased yield in continuous ginseng cultivation, changes in soil fungal communities are a major factor (Zhu et al., 2020). Researchers have found that the richness and diversity of rhizosphere fungi can significantly affect the yield and quality of crops (Gao et al., 2019; Sun et al., 2022), which supports the above finding. However, there is still no systematic understanding of the specific effects of different root exudate concentrations on the yield and quality of ginseng, and on the rhizosphere fungal communities. Carrying out an integrated study of the relationship between root exudates and soil fungal communities is not only important for exploring soil microbial diversity in ginseng fields and controlling soil-borne diseases in ginseng, but it will also greatly contribute to our understanding of the feedback behaviour in plant-soil systems (Cai et al., 2021).

In summary, to clarify the ginseng-root exudates-rhizosphere fungi relationships, we explored the effects of different root exudate concentrations on ginseng growth indexes (plant height, stem and leaf fresh weight, rhizome fresh weight, and rhizome dry weight), medicinal ginsenoside levels, and rhizosphere fungal communities. The main objectives of the study were to: (i) reveal the trends in ginseng growth indexes under different root exudate concentrations; (ii) elucidate the characteristics of the ginseng rhizosphere fungal community structure under different root exudate concentrations; and (iii) explore the responses of medicinal ginsenoside levels to changes in the ginseng rhizosphere fungal community structure. On this basis, we hope to develop a new ecological strategy to alleviate ginseng soil sickness by regulating the root exudate concentration and the rhizosphere fungal community.

## 2. Materials and methods

### 2.1. Preparation and composition analysis of ginseng root exudates

The extraction of root exudates was carried out according to the method established by Williams et al. (2021), with slight modification. Sand:perlite at a volume ratio of 3:2 was used as a soilless culture matrix. Three-years-old ginseng was rinsed with sterile water and planted in this substrate. The surface of the substrate was covered with thin cotton to prevent impurities from falling into the substrate. During the growth of ginseng, distilled water and nutrient solution (Hoagland’s nutrient solution, mainly composed of potassium nitrate, calcium nitrate, magnesium sulfate, phosphate, etc.) were alternately used for irrigation once a week. In mid-October, the plants were pulled out and the substrates were collected. The root exudates were extracted using ultrapure water and 95% ethanol. The extraction was carried out at a solid-to-liquid ratio of 1:20 (w/v), using constant-temperature shaking (28°C, 24 h) followed by ultrasonic extraction (50 Hz, 25°C, 30 min). It was repeated three times. The extracts were combined, centrifuged (5,000 g, 10 min), and filtered through a 0.22 μm membrane. The filtrate was dried under reduced pressure and stored at −20°C.

The analysis of the root exudate composition referred to the gas chromatography (GC)-mass spectrometry (MS) method established by Xiang et al. (2022). A small sample of the extract was dissolved in methanol and passed through a 0.22 μm filter membrane. Subsequently, a GC-MS system (Agilent 7000D gas chromatograph and 8,890 mass spectrometer; Agilent Technologies, Santa Clara, United States) was used to analyze the root exudate composition. The capillary column was HP-5MS (30.0 m × 0.25 mm × 0.25 μm), the carrier gas was He, the flow rate was 1 mL min^−1^, the injection volume was 1 μL, and the inlet temperature was 250°C. The measurement procedure involved the following: the column temperature was 45°C, held for 2 min, 5°C min^−1^ to 280°C, held for 10 min. The MS conditions were as follows: electron ionization (EI) source, 70 eV; scan range, 20–800 amu. Qualitative and quantitative analyses were performed by comparing the data obtained from the GC-MS analysis with the NIST20.L spectral library and retaining the components with a match >80%.

### 2.2. Site description and experiment design

The experimental site was located in Qixing Baicao Garden, Changchun City, Jilin Province, China (43°49′48″ N, 125°24′53″ E). The area is 399 m above sea level, with a mean annual temperature of 4.6°C and annual precipitation of about 600 to 700 mm. In the experiment, 500 g of farmland soil was placed in 8 × 8 cm pots. Two-years-old ginseng plants of uniform size and good growth were transplanted (one plant per pot). The soil was dark brown sandy loam, with pH 6.39, organic matter 51.56 g·kg^−1^, available nitrogen 31.06 mg·kg^−1^, available phosphorus 28.63 mg·kg^−1^, and available potassium 86.33 mg·kg^−1^.

In June 2021, six different concentrations of ginseng root exudates (total organic carbon 7.93 mg·g^−1^) were added to the potted soil by dissolving appropriate amounts of ginseng root exudates in a constant volume of water and evenly spraying the water onto the soil surface of the pots. The added root exudate concentrations in the six groups (with six replicate pots per group) were 0 mg·g^−1^ (CK), 0.3 mg·g^−1^ (T1), 1.5 mg·g^−1^ (T2), 3 mg·g^−1^ (T3), 6 mg·g^−1^ (T4), and 15 mg·g^−1^ (T5). Other factors (such as protective measures, irrigation, and weeding) during the cultivation period were consistent with field factors to ensure that the external differences among the treatments were minimized.

### 2.3. Sample collection and growth index determination

After 60 days of cultivation, the ginseng plants and rhizosphere soil in each treatment group were collected. The plant growth indexes (plant height, stem and leaf fresh weight, rhizome fresh weight, and rhizome dry weight) were then measured (six replicates per group). The surface soil was removed from the rhizomes and the rhizosphere soil was then collected with a brush and mixed, and divided into three equal parts per group of samples. The non-medicinal parts of the plants were then removed and the rhizomes were cleaned, dried, ground into fine powder, sieved (250 μm), and divided into three equal parts per group of samples (for ginsenoside level determination). The samples were immediately stored in a refrigerator at −80°C prior to analysis.

### 2.4. Determination of the levels of 10 ginsenosides in the rhizomes

The levels of ginsenosides (saponins) Rg1 (C_42_H_72_O_14_, CAS: 22427-39-0), Re (C_48_H_82_O_18_, CAS: 51542-56-4), Rf (C_42_H_72_O_14_, CAS: 52286-58-5), Rg2 (C_42_H_72_O_13_, CAS: 52286-74-5), Rb1 (C_54_H_92_O_23_, CAS: 41753-43-9), Ro (C_48_H_76_O_19_, CAS: 34367-04-9), Rc (C_53_H_90_O_22_, CAS: 11021-14-0), Rb2 (C_53_H_90_O_22_, CAS: 11021-13-9), Rb3 (C_53_H_90_O_22_, CAS: 68406-26-8), and Rd. (C_48_H_82_O_18_, CAS: 52705-93-8) in 18 rhizomes samples (3 per group) were determined using a high-performance liquid chromatography (HPLC)-UV detector (Shimadzu, Kyoto, Japan). The specific assay conditions referred to the method established in the previous phase of this study (Sun et al., 2022).

### 2.5. DNA extraction, PCR amplification, and illumina sequencing

The soil fungal community analysis referred to the method of Sun et al. (2022), with slight modification. An MN NucleoSpin Soil DNA Kit (Macherey-Nagel, Germany) was used to extract the total rhizosphere soil DNA from 18 soil samples (3 per group). The quality and concentration of DNA were assessed using 1.8% (w/v) agarose gel electrophoresis and a Multiskan Sky full-wavelength microplate reader (Thermo Fisher Scientific, Waltham, MA, United States). A soil fungal *ITS* full-length PCR amplification system, with primers *ITS1F* (5′-CTT GGT TTA GAG GAA GTA A-3′) and *ITS2* (5′-GCT GCG TTC TTC ATC GAT GC-3′), was used for amplification. The reaction system comprised template DNA 50 ng, KOD FX Neo 0.2 μL, KOD FX Neo buffer 5 μL, 2 mM dNTP 2 μL, 10 μM forward and reverse primers 0.3 μL, and ddH_2_O to 10 μL. The reaction procedure was as follows: pre-denaturation at 95°C for 5 min, 25 cycles of 95°C for 30 s, 50°C for 30 s, and 72°C for 40 s, and a final extension at 72°C for 5 min. The PCR products were quantified using a microplate reader and mixed according to the mass ratio of 1:1. After mixing, an OMEGA DNA purification column was used for purification. Sequencing was then performed on an Illumina HiSeq platform using standard protocols.

### 2.6. Sequencing data processing and statistical analysis

Raw reads were filtered with Trimmomatic-0.33, then Cutadapt (v 1.9.1) was used to identify and remove primer sequences to obtain clean reads. The clean reads for each sample then underwent splicing and length filtering using Usearch (v 10). The Effective Reads were obtained by removing chimeric sequences using UCHIME (v 4.2). Sequences with 97% nucleotide identity were then clustered into operational taxonomic units (OTUs) using Usearch (v 10.0). A representative sequence from each OTU was classified using the UNITE database (v 7.2, http://unite.ut.ee/index.php).

Subsequently, community richness and diversity indices were calculated using QIIME (v 1.9.1). Dilution curves were plotted using R software (v 3.6.0), and Bray–Curtis distance tests were performed for differences in community composition. Principal coordinate analysis (PCoA) as performed using the WGCNA, stat, and ggplot2 packages in R. Heatmaps were generated using the Vegan package in R to analyze the composition of the fungal communities.

Statistical analysis and graphical plotting of ginseng growth indexes and ginsenoside levels were performed using SPSS (v 21.0), GraphPad Prism (v 8.0), and Origin (v 2019). All values are expressed as mean ± standard deviation (SD). Normality of distribution and homogeneity of variance were evaluated before the statistical analysis was conducted. One-way analysis of variance (ANOVA) with student’s *t*-test was used for statistical comparison, and *p* < 0.05 was considered statistically significant.

## 3. Results

### 3.1. GC-MS analysis of ginseng root exudates

The extraction rate of root exudate from the rhizosphere soil of 3 years-old ginseng was 0.38%. GC-MS was used to identify the chemical components of the exudate (Supplementary Figure S1). Twenty of the major chemical components were isolated and identified (Table 1).

**Table 1:** Component analysis of ginseng root exudates.

Number	RT time	Compound	CAS	Content (%)
1	4.15	Acetic acid	64-19-7	0.23%
2	10.406	Benzaldehyde	100-52-7	0.38%
3	15.6	Methyl phenylacetate	101-41-7	0.27%
4	17.725	3,4-Dimethyl-benzaldehyde	5973-71-7	0.49%
5	18.551	2-Hydroxy-iso-butyrophenone	7473-98-5	0.09%
6	21.627	Vanillin	121-33-5	0.11%
7	23.057	Dimethyl phthalate	131-11-3	0.83%
8	25.994	2,4-Di-tert-butylphenol	96-76-4	1.18%
9	29.043	Diisobutyl adipate	141-04-8	1.58%
10	30.41	Methyl 10-methyl-hexadecanoate	1000336-50-9	18.61%
11	32.03	Methyl palmitate	112-39-0	8.36%
12	34.245	3,7-Dihydroxy-3-phenyl-4-chromanone	95296-98-3	20.65%
13	36.411	Oleic acid	112-80-1	0.41%
14	36.758	Methyl trans-9-octadecenoate	1937-62-8	1.26%
15	40.007	Hexadecanamide	629-54-9	3.23%
16	41.306	N-ethyl-myristamide	1000408-01-8	6.45%
17	42.276	Oleamide	301-02-0	1.71%
18	44.252	Bis(2-ethylhexyl) adipate	103-23-1	3.25%
19	47.605	Pisiferanol	99152-14-4	0.13%
20	57.822	Tigogenin	77-60-1	1.14%

### 3.2. Effects of root exudate concentrations on growth indexes of ginseng

Biological characteristics such as growth indexes (Table 2) visually reflect the adaptability of plants to the soil environment and are important indicators for evaluating plant vigor. Low root exudate concentrations (T1 and T2) had little effect on plant height, but plant height decreased with further increases in the root exudate concentration, with the largest decrease in the T4 group (*p* < 0.05), which was 23.78% lower than in the CK group. Similarly, the lowest stem and leaf fresh weight occurred in the T4 group and there was no significant difference between T5 and T4 (*p* > 0.05). Interestingly, fresh and dry rhizome weights were higher in the low root exudate concentration groups (T1 and T2) than the CK group but significantly lower (*p* < 0.05) in the high root exudate concentration groups (T4 and T5) than the CK group, and lowest under T5 (40.09% and 38.35%, respectively, lower than in the CK group).

**Table 2:** Effects of different root exudate concentrations on growth indexes of ginseng (*n* = 6).

Treatment	Plant height (cm)	Stem and leaf fresh weight (g)	Rhizome fresh weight (g)	Rhizome dry weight (g)
CK	73.30 ± 3.90^a^	6.40 ± 0.59^a^	9.13 ± 1.83^a^	2.06 ± 0.27^ab^
T1	72.13 ± 2.99^a^	6.13 ± 0.55^a^	9.56 ± 1.50^a^	2.20 ± 0.32^ab^
T2	73.77 ± 4.97^a^	5.98 ± 0.64^a^	9.70 ± 1.05^a^	2.33 ± 0.40^a^
T3	65.57 ± 8.63^ab^	5.47 ± 0.51^a^	7.26 ± 1.17^ab^	1.85 ± 0.32^abc^
T4	55.87 ± 7.66^b^	3.95 ± 0.57^b^	5.61 ± 1.39^b^	1.53 ± 0.27^bc^
T5	59.40 ± 6.66^ab^	4.03 ± 0.24^b^	5.47 ± 1.02^b^	1.27 ± 0.17^c^

Different letters indicate significant differences between treatments, *p* < 0.05. Root exudate concentrations: CK, 0 mg·g^−1^; T1, 0.3 mg·g^−1^; T2, 1.5 mg·g^−1^; T3, 3 mg·g^−1^; T4, 6 mg·g^−1^; T5, 15 mg·g^−1^.

### 3.3. Effects of root exudate concentrations on 10 ginsenosides in ginseng

**Figure 1:**
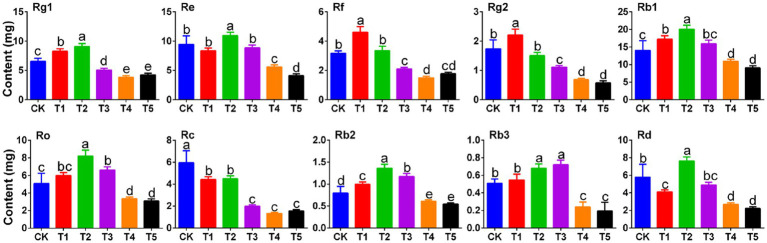
Effects of root exudate concentrations on ginsenoside levels in ginseng rhizomes (*n* = 3). Different letters indicate significant differences between treatments, *p* < 0.05. Root exudate concentrations: CK, 0 mg·g^−1^; T1, 0.3 mg·g^−1^; T2, 1.5 mg·g^−1^; T3, 3 mg·g^−1^; T4, 6 mg·g^−1^; T5, 15 mg·g^−1^.

The levels of 10 major ginsenosides were determined by analyzing the concentration of each individual ginsenoside (Supplementary Figure S2) in ginseng rhizomes treated with different root exudate concentrations. As shown in Figure 1, the responses of each ginsenoside to different root exudate concentrations differed.

Low root exudate concentration promoted the synthesis of Rg1, which peaked under T2 (1.39 times that in the CK group). However, Rg1 decreased with further increases in the root exudate concentration (35.23% decrease in the T5 group compared to the CK group). The trends in Ro, Rb1, Rb2, Re, and Rd. were similar to Rg1; when the root exudate concentration was 1.5 mg·g^−1^ (T2), each ginsenoside (Ro, Rb1, Rb2, Re, and Rd) was significantly higher than in the CK group (*p* < 0.05) but when the root exudate concentration reached 15 mg·g^−1^ (T5), each ginsenoside was lowest. Unlike Rg1, Rf and Rg2 peaked under T1 and then decreased with increasing root exudate concentrations. Rb3 peaked under T3 (1.41 times that in the CK group) and was lowest under T5 (significantly lower than in the CK group (*p* < 0.05)). Increasing root exudate concentrations did not promote the accumulation of Rc, which tended to decrease gradually with increasing root exudate concentrations.

### 3.4. Effects of root exudate concentrations on proportions and total absolute level of 10 ginsenosides in ginseng

**Figure 2:**
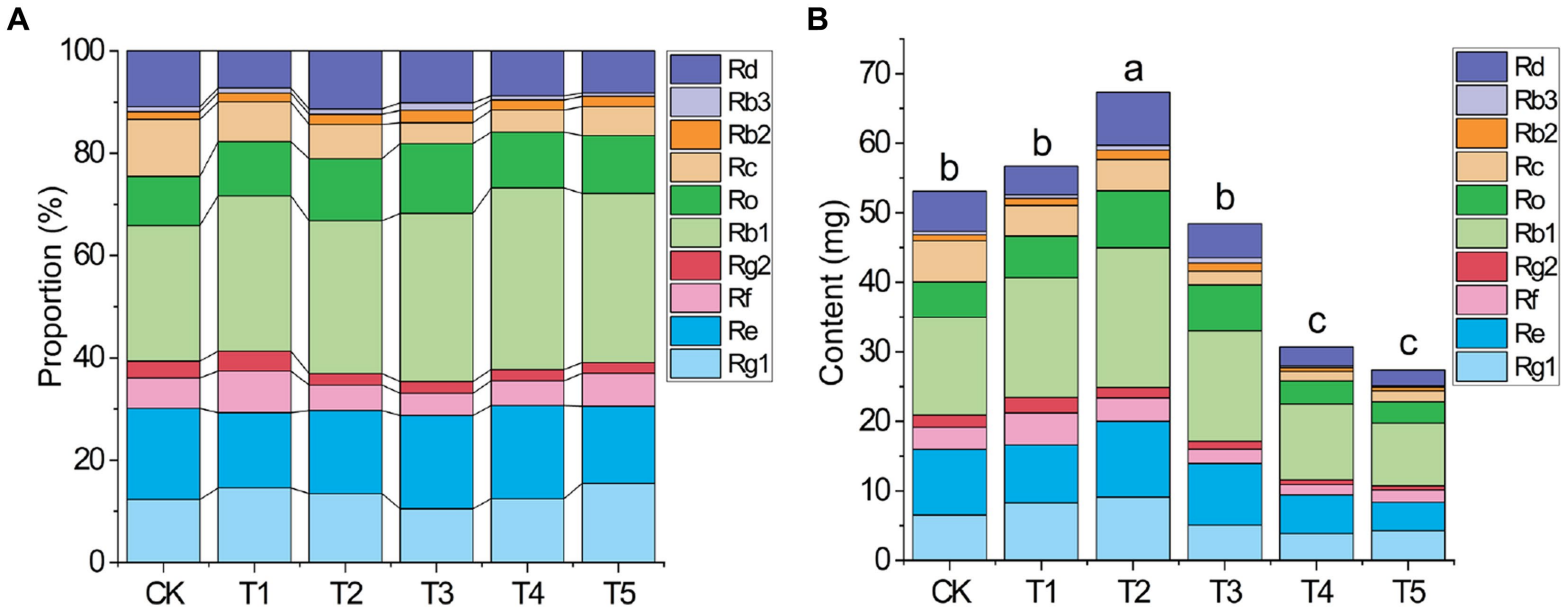
Effects of root exudate concentrations on the proportion of each ginsenoside **(A)** and absolute level of all 10 ginsenosides **(B)** in ginseng (*n* = 3). Different letters indicate significant differences between treatments, *p* < 0.05. Root exudate concentrations: CK, 0 mg·g^−1^; T1, 0.3 mg·g^−1^; T2, 1.5 mg·g^−1^; T3, 3 mg·g^−1^; T4, 6 mg·g^−1^; T5, 15 mg·g^−1^.

The quality of medicinal herbs depends on the proportions and total absolute level of medicinal components. Under different root exudate concentrations, the proportion of each ginsenoside among the 10 ginsenosides changed (Figure 2A and Supplementary Table S1). For example, in the T2 group, the proportions of Ro and Rb1 increased and the proportions of Rc and Re decreased compared to the CK group.

The change in the total absolute ginsenoside level (Figure 2B) under different root exudate concentrations was similar to that of the rhizome biomass (Table 2). The total absolute ginsenoside level increased when the root exudate concentration was ≤1.5 mg·g^−1^ (T2), peaking in the T2 group (1.27 times that in the CK group). When the root exudate concentration was >1.5 mg·g^−1^, it gradually decreased with increasing root exudate concentration, being lowest in the T5 group (only 51.56% of that in the CK group).

### 3.5. Effects of root exudate concentrations on fungal community *α*-diversity

High-throughput sequencing was performed on 18 soil samples using an Illumina HiSeq platform (Table 3). The dilution curves showed that the number of OTUs of the samples was close to the saturation level, indicating that there were enough samples to perform the assay and the sequencing was sufficient to reflect the real situation of the samples (Supplementary Figure S3). The number of clean reads per sample ranged from 61,759 to 79,729 and the number of OTUs ranged from 1,525 to 1,742, and the coverages of all samples were >99.70%.

**Table 3:** High-throughput sequencing results and *α*-diversity indexes of ginseng rhizosphere soil samples (*n* = 3).

Treatment	Clean reads	OTUs	Coverage	ACE	Chao1	Shannon	Simpson
CK	79,722	1,595 ± 68	0.999	1,613 ± 67^a^	1,626 ± 70^a^	8.37 ± 0.07^a^	0.9873 ± 0.0007^b^
T1	79,621	1,594 ± 65	0.999	1,613 ± 66^a^	1,622 ± 65^a^	8.35 ± 0.01^a^	0.9871 ± 0.0002^b^
T2	79,541	1,598 ± 74	0.999	1,617 ± 79^a^	1,628 ± 81^a^	8.34 ± 0.03^a^	0.9869 ± 0.0004^b^
T3	79,647	1,614 ± 116	0.999	1,634 ± 109^a^	1,645 ± 103^a^	8.36 ± 0.2^a^	0.9881 ± 0.002^b^
T4	79,729	1,742 ± 8	0.999	1,765 ± 6^a^	1,783 ± 6^a^	8.57 ± 0.02^a^	0.9908 ± 0.0006^a^
T5	61,759	1,525 ± 84	0.997	1,596 ± 66^a^	1,609 ± 51^a^	8.4 ± 0.13^a^	0.9918 ± 0.0007^a^

Different letters indicate significant differences between treatments, *p* < 0.05. Root exudate concentrations: CK, 0 mg·g^−1^; T1, 0.3 mg·g^−1^; T2, 1.5 mg·g^−1^; T3, 3 mg·g^−1^; T4, 6 mg·g^−1^; T5, 15 mg·g^−1^.

The ACE and Chao1 indexes evaluate the species richness of the samples, and the Shannon and Simpson indexes evaluate the species diversity of the samples. Analysis of the ginseng rhizosphere fungal communities showed that the ACE and Chao1 indices showed similar trends under increasing root exudate concentrations, first increasing and then decreasing, peaking under T4. Similarly, the Shannon index peaked under T4. Unlike the Shannon index, the Simpson index peaked under T5, which was significantly higher than that in the CK group (*p* < 0.05).

### 3.6. Effects of root exudate concentrations on fungal community composition

**Figure 3:**
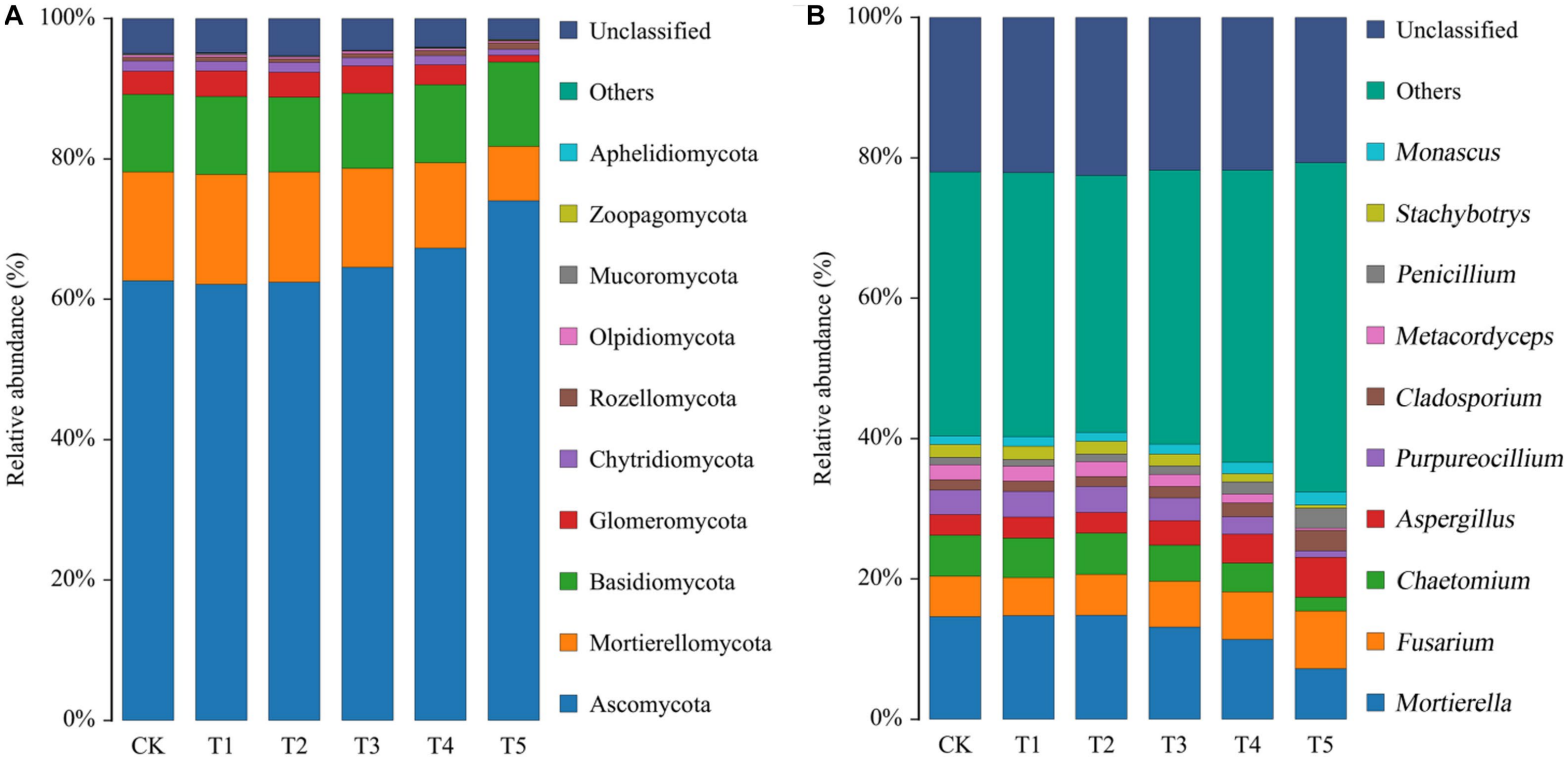
Relative abundances of fungal phyla **(A)** and genera **(B)** in the rhizosphere soil of ginseng under different root exudate concentrations (*n* = 3). Root exudate concentrations: CK, 0 mg·g^−1^; T1, 0.3 mg·g^−1^; T2, 1.5 mg·g^−1^; T3, 3 mg·g^−1^; T4, 6 mg·g^−1^; T5, 15 mg·g^−1^.

The fungal taxa were identified on the basis of OTUs (Figure 3). At the phylum level (Figure 3A), the fungi in ginseng rhizosphere soil were mainly *Ascomycota*, *Mortierellomycota*, and *Basidiomycota*. The relative abundances of the top 10 fungal phyla are listed in Supplementary Table S2. There were no notable differences in the relative abundance of each dominant phylum between the low root exudate concentration groups (T1 and T2) and the CK group, but the differences after further increases in the root exudate concentration were notable. Among the phyla, the relative abundance of *Ascomycota* increased with increasing root exudate concentration, peaking under T5 (relative abundance, 74.04%). Unlike Ascomycota, the relative abundances of *Mortierellomycota* and *Glomeromycota* decreased with increasing root exudate concentration, being lowest under T5 (50.06 and 29.00% of that in the CK group).

The top 10 fungal genera are shown in Figure 3B, and their relative abundances are listed in Supplementary Table S3. As with the phyla, there was no significant difference in the relative abundance of each dominant fungal genus between the treatment groups and the CK group when root exudate concentrations were ≤1.5 mg·g^−1^. However, the relative abundance of each fungal genus changed considerably with increasing root exudate concentrations. Among them, *Mortierella*, *Chaetomium*, *Purpureocillium*, *Metacordyceps*, and *Stachybotrys* gradually decreased with increasing root exudate concentrations compared to the CK group. However, the opposite was true for *Fusarium*, *Aspergillus*, *Cladosporium*, *Penicillium*, and *Monascus*, as they significantly increased compared to the CK group when the root exudate concentration increased. These five genera peaked under T5 (1.43, 1.93, 1.97, 2.62, and 1.56 times that in the CK group, respectively).

### 3.7. Effects of root exudate concentrations on fungal community structure

**Figure 4:**
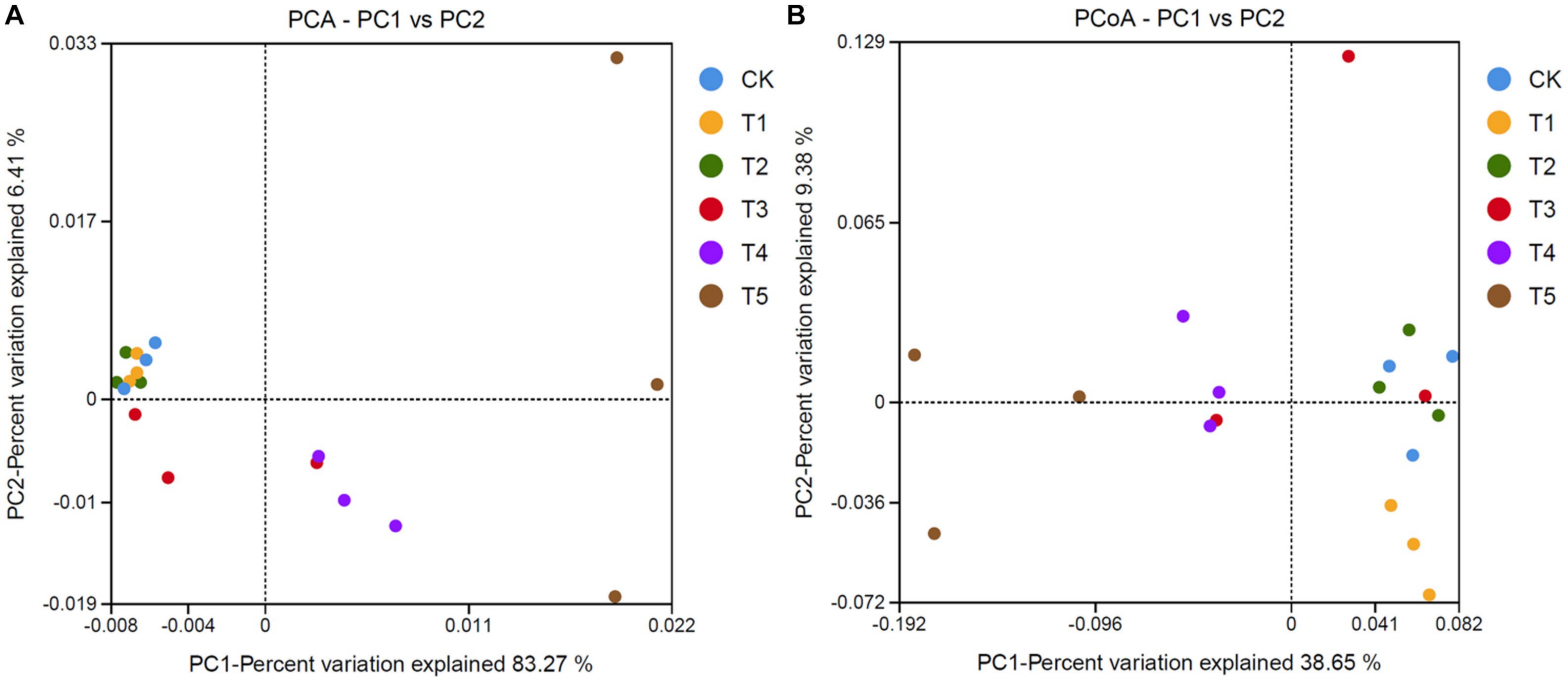
Analysis of fungal community structure in ginseng rhizosphere soil. **(A)** PCA and **(B)** PCoA of fungal communities based on OTUs. Root exudate concentrations: CK, 0 mg·g^−1^; T1, 0.3 mg·g^−1^; T2, 1.5 mg·g^−1^; T3, 3 mg·g^−1^; T4, 6 mg·g^−1^; T5, 15 mg·g^−1^.

To compare the community structure of ginseng rhizosphere fungi under different root exudate concentrations, PCA, PCoA, and analysis of similarities (ANOSIM) were carried out. PCA (Figure 4A) showed that the two main extracted coordinates cumulatively explained 82.21% of the variance, with PC1 and PC2 explaining 83.27 and 6.41%, respectively. The T1 and T2 groups were less different from the CK group, while the T3, T4, and T5 groups gradually moved to the right along the PC1 direction as the root exudate concentration increased. PCoA (Figure 4B) also showed differences in fungal community structure under different root exudate concentrations. The T1 and T2 groups were on the same side of the coordinate axis as the CK group, while the T4 and T5 groups were on the other side. Also, ANOSIM showed moderate differences (*R* = 0.556, *p* = 0.001) in rhizosphere fungal communities under different root exudate treatment concentrations (Supplementary Figure S4).

**Figure 5:**
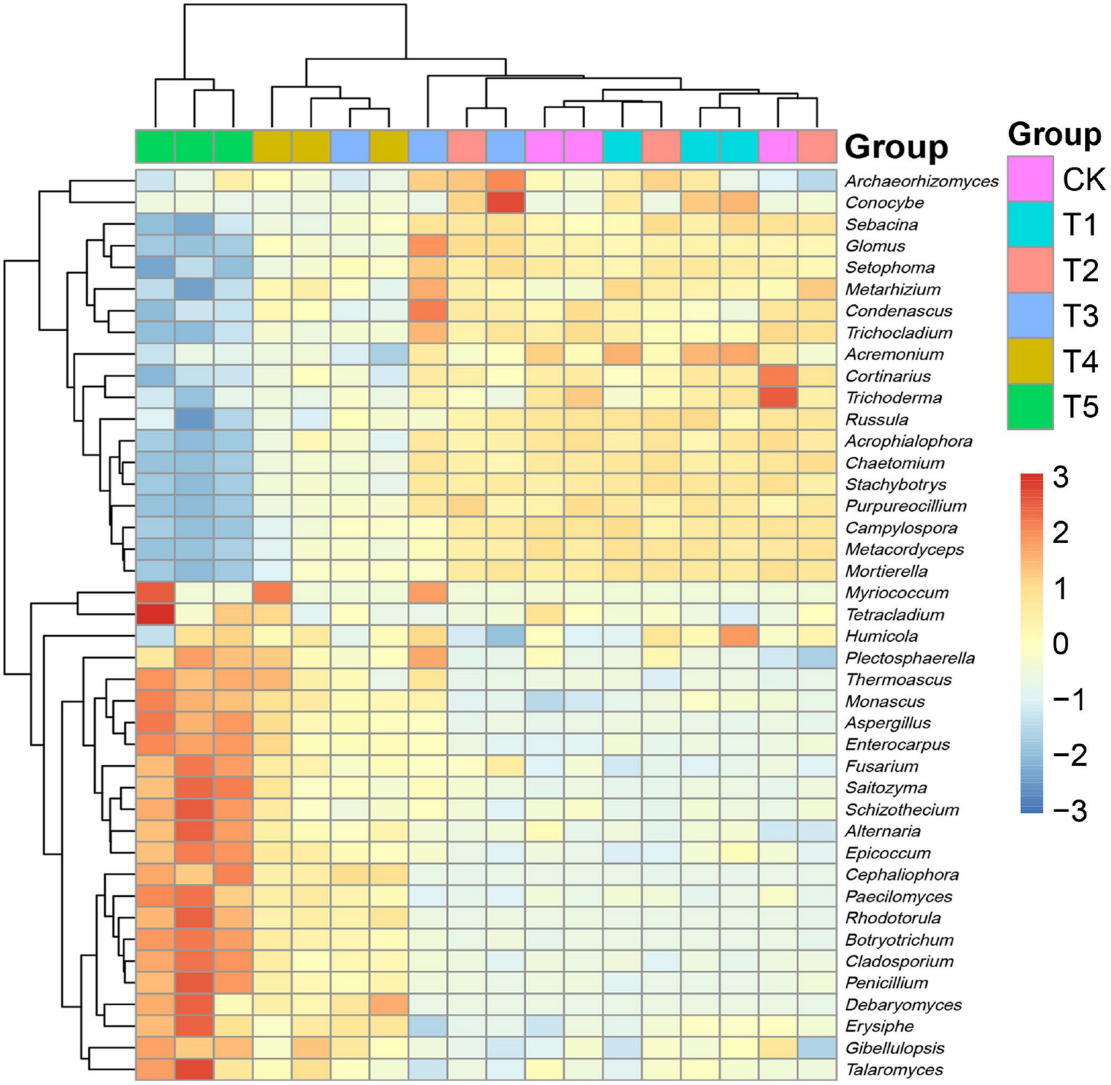
Heatmap of relative abundances of fungal genera. Root exudate concentrations: CK, 0 mg·g^−1^; T1, 0.3 mg·g^−1^; T2, 1.5 mg·g^−1^; T3, 3 mg·g^−1^; T4, 6 mg·g^−1^; T5, 15 mg·g^−1^.

To clarify the structural differences among the fungal communities under different root exudate concentrations, we constructed a relative abundance heatmap for genera with relative abundance >5% (Figure 5). The results showed that the low root exudate concentration groups (T1 and T2) clustered with the CK group, while the fungal communities differed more between the high concentration groups (T4 and T5) and the CK group, which was consistent with the results of PCA and PCoA.

**Figure 6:**
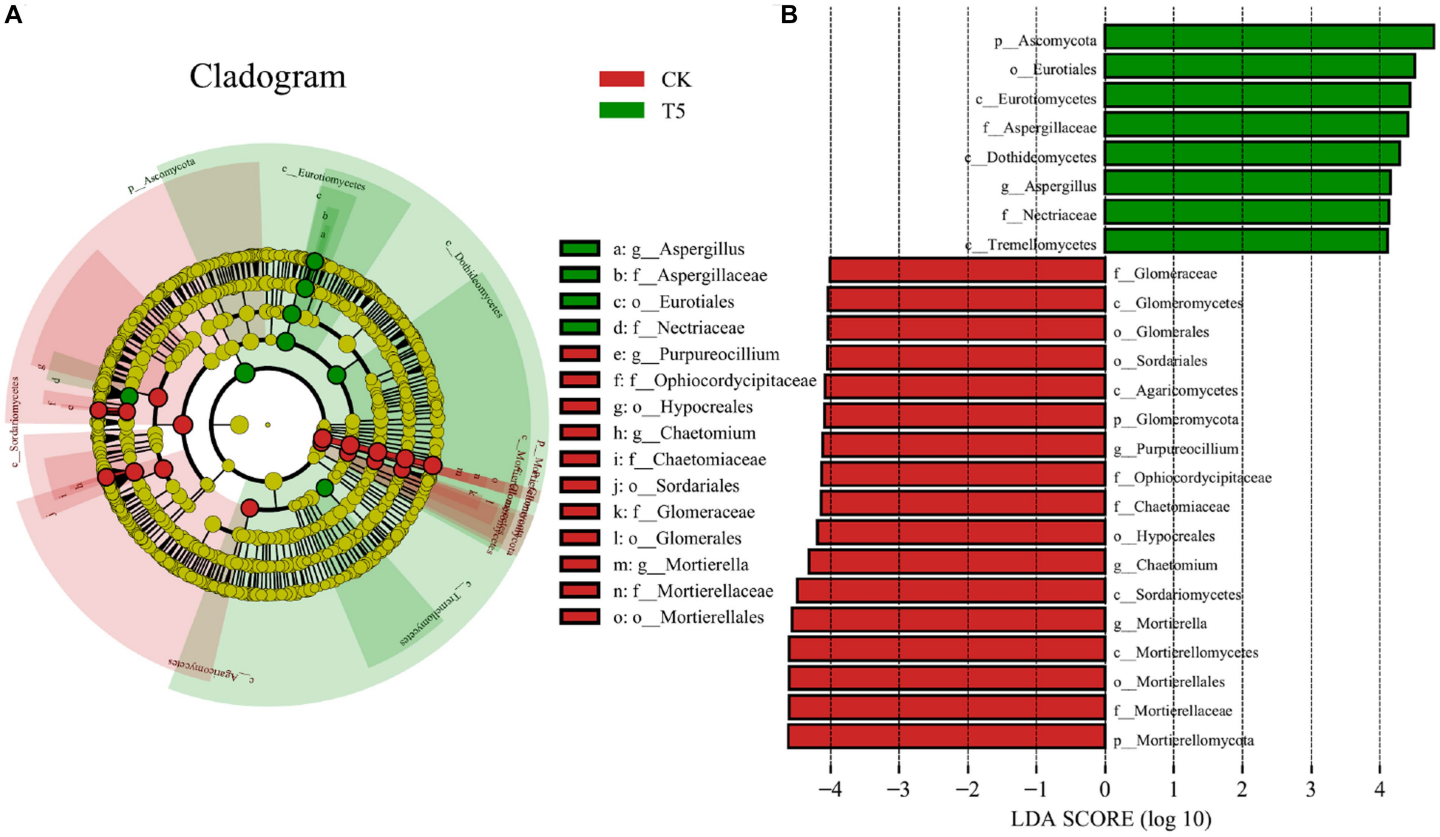
LEfSe analysis of rhizosphere fungal communities in the CK and T5 groups. **(A)** Cladogram of taxonomic levels from phylum to genus and **(B)** taxa with LDA values > 4. Root exudate concentrations: CK, 0 mg·g^−1^; T5, 15 mg·g^−1^.

To further clarify the structural differences in the rhizosphere fungal communities in the high root exudate concentration group (T5) compared to the CK group, we performed LEfSe analysis for the T5 and CK groups and explored the biomarkers from phyla to genera (Figure 6). The representative phyla of the CK group were *Mortierellomycota* and *Glomeromycota*, and that in the T5 group was Ascomycota. At the class and order levels, *Eurotiomycetes* (class), *Dothideomycetes* (class), *Tremellomycetes* (class), *Eurotiales* (order), *Aspergillaceae* (order) and *Nectriaceae* (order) were representative for the T5 group. The representative genera of the CK group were *Mortierella*, *Chaetomium*, and *Purpureocillium* and that in the T5 group was *Aspergillus*.

### 3.8. Correlations among 10 major rhizosphere fungal genera and yield and quality of ginseng

**Figure 7:**
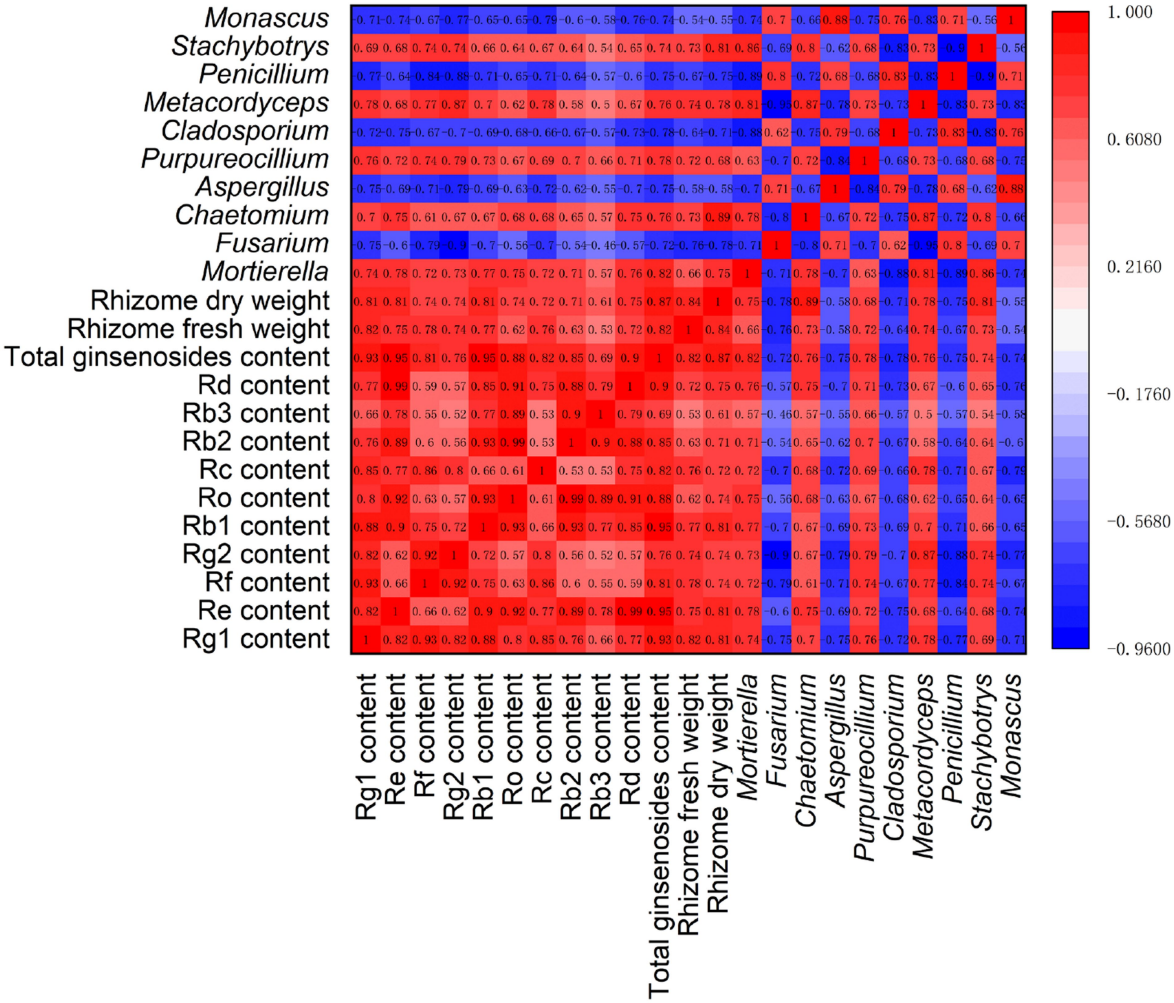
Correlation analysis of ginseng yield, quality, and fungal genera under different root exudate concentrations.

Spearman correlation analysis was performed, involving ginseng yield (dry and fresh weight of rhizomes), quality (ginsenoside levels), and rhizosphere fungal communities (relative abundances of 10 major fungal genera) (Figure 7). The results showed that there were significant positive correlations (*p* < 0.05) among the ginsenosides in ginseng rhizomes. However, there were large differences in the correlations among the 10 major fungal genera. Among them, there were significant positive correlations (*p* < 0.05) among *Fusarium*, *Aspergillus*, *Cladosporium*, *Penicillium*, and *Monascus*. However, they were significantly negatively correlated (*p* < 0.05) with five other fungal genera (*Mortierella*, *Chaetomium*, *Purpureocillium*, *Metacordyceps*, and *Stachybotrys*), suggesting that there may be a strong antagonistic effect between the two fungal subgroups.

Notably, the relative abundances of the fungal genera *Mortierella*, *Chaetomium*, *Purpureocillium*, *Metacordyceps*, and *Stachybotrys* were significantly positively correlated with each ginsenoside and the total ginsenoside level, with correlation coefficients >0.50 (*p* < 0.05). Similarly, these five genera were significantly positively correlated with the fresh and dry weight of rhizomes, indicating that these genera may promote ginseng rhizome growth and the accumulation of medicinal ginsenosides. The opposite was true for the fungal genera *Fusarium*, *Aspergillus*, *Cladosporium*, *Penicillium*, and *Monascus*, which were significantly negatively correlated (*p* < 0.05) with both ginsenoside levels and rhizome biomass, suggesting that these fungal genera may exert inhibitory effects on ginseng rhizome growth and ginsenoside synthesis.

## 4. Discussion

### 4.1. Effects of root exudate concentrations on the growth and quality of ginseng

Plants release a series of compounds into the soil through their roots during the growth phase. These compounds, which are called root exudates (Li et al., 2022), determine the interactions between plants and their surroundings. Therefore, a thorough understanding of plant root exudates is important for basic science as well as for improving crop yield and quality (Escolà Casas and Matamoros, 2021). This study showed that the main components of ginseng root exudates were amides, organic acids, and esters. Each growth index of ginseng (plant height, stem and leaf fresh weight, rhizome fresh weight, and rhizome dry weight) showed that the plants had better adaptability at root exudate concentrations ≤1.5 mg·g^−1^ (T2). However, with further increases in the root exudate concentration, all growth indexes were inhibited. This was especially the case for the rhizome dry weight (which was significantly reduced compared to in the CK group), which determines the yield of ginseng. This highlights that the root exudate concentration is an important factor in regulating the growth and development of ginseng. Excitingly, previous research on other plants of the same family, such as *Panax notoginseng* and *Panax quinquefolium*, showed that these plants had similar responses to the effect of high root exudate concentrations, specifically apoptosis of root tip cells and inhibition of seedling germination and growth, suggesting that the accumulation of root exudates may disrupt soil homeostasis, negatively affect crop growth, and consequently reduce crop yield (He et al., 2009; Yang et al., 2015).

The cultivation of medicinal herbs should not only consider yield but also focus on quality (Ostadi et al., 2020). This study showed that appropriately low root exudate concentrations promoted the accumulation of multiple ginsenosides, though the intensity of the response of each of these ginsenosides to the root exudate concentration changes differed. Six of the ginsenosides (Rg1, Ro, Rb1, Rb2, Re, Rd) peaked under T2 (added root exudate concentration of 1.5 mg·g^−1^), while Rf and Rg2 peaked under T1. However, with further increases in the root exudate concentration, each ginsenoside decreased, being significantly lower under T5 than in the CK group. Interestingly, increasing root exudate concentrations did not promote the accumulation of Rc, which tended to decrease gradually with increasing root exudate concentrations. Subsequently, a comprehensive analysis of the total ginsenoside level (Figure 2) also showed that low root exudate concentrations increased this level, but further increases in the root exudate concentration had a significant negative impact. Therefore, we speculate that there are many allelopathic/autotoxic substances in ginseng root exudates, and their accumulation drives changes in rhizosphere abiotic or biotic characteristics, resulting in plant-soil negative feedback. It is worth noting that the regulation of crops by root exudates seems to involve complex processes that are entirely dependent on the feedback behavior of the plant-soil system (Luo et al., 2022). A deeper understanding of this feedback behavior is necessary for optimum ecological conservation and agricultural development.

### 4.2. Effects of root exudate concentrations on the rhizosphere fungal community of ginseng

Root exudates are important mediators of material exchange and information exchange in plant inter-root ecosystems, not only directly affecting soil productivity and plant growth, but also playing an important regulatory role regarding microbial diversity and community composition (Coskun et al., 2017; Wu et al., 2018). The *α*-diversity analysis (Table 3) showed an overall increasing and then decreasing trend in the richness and diversity of ginseng rhizosphere fungi with increasing root exudate concentrations. This provides a prerequisite for the regulation of fungal communities through root exudates to improve soil function and thus maintain plant health (Guo et al., 2018). The composition and abundance of fungal communities play important roles in the soil microecological balance (Wang et al., 2021). The species abundance heatmap (Figure 3) showed that Ascomycota was the dominant fungal phylum in ginseng rhizosphere soil, and its relative abundance gradually increased with increasing root exudate concentrations. The second most dominant phylum was *Mortierellomycota*, which is a biomarker in the rhizosphere soil of high-quality ginseng and is considered to be closely related to soil nutrients (Fang et al., 2022). The relative abundance of *Mortierellomycota* significantly decreased when the root exudate concentration was high, indicating that the accumulation of root exudates may suppress potentially beneficial fungi and thus affect plant health. Regarding the genera, the relative abundance of each fungal genus did not change significantly compared to the CK group when the root exudate concentration was low (added at ≤1.5 mg·g^−1^). When the root exudate concentration was higher, the relative abundance of potentially beneficial fungi decreased. Among them, *Mortierella* and *Chaetomium* are eutrophic fungi that can rapidly access simple carbon sources, which makes them good candidates to compete with fast-growing pathogenic fungi for nutrients (Zhao et al., 2018; Ma et al., 2021). *Purpureocillium*, *Metacordyceps*, and *Stachybotrys* can be used for biological control and have important roles in the control of insects and nematodes, among others (Elsherbiny et al., 2021). Obviously, the decrease in the abundance of potential beneficial fungi in the ginseng rhizosphere is likely to lead to an increase in pathogenic fungi, which seriously endangers plant growth and development. Predictably, the relative abundance of potentially pathogenic fungi gradually increased with increasing root exudate concentrations. Among these potentially pathogenic fungal genera, *Fusarium*, *Aspergillus*, and *Penicillium* are the main causes of ginseng root rot, resulting in decreased yield and poor growth (Li et al., 2018; Du et al., 2022). The fungal genera *Cladosporium* and *Monascus* are major causes of ginseng wilt, which can cause leaf scorch and even plant death (Goodwin, 2022). This further suggests that the imbalance of rhizosphere microecology caused by the massive accumulation of ginseng root exudates may be an important factor underlying the reduced yield and quality of ginseng. Excitingly, several recent studies have reported similar observations. For example, root exudates (phenolic acids) were shown to alter the rhizosphere fungal community structure of ginseng, increase the fungal pathogenic load, lead to root rot, and limit plant growth (Li et al., 2018). In addition, a study on *Panax notoginseng* showed that when the root exudates reached a certain concentration, significant autotoxicity was produced, resulting in replanting failure (Xiang et al., 2022).

Fungal community structure analysis (Figures 4, 5) showed that the structure of the ginseng rhizosphere fungal community was similar to that in the CK group at low root exudate concentrations (T1 and T2), but it varied more at higher concentrations (T4 and T5). To clarify the changes in fungal community structure at higher concentrations, we performed LEfSe analysis for the CK and T5 groups (Figure 6). The biomarker genera indicated that a high root exudate concentration (T5) increased the pathogenic fungi and left the fungal community devoid of multiple ecosystem functions. This highlights the fact that root exudates can selectively modulate the abundances of certain core microbial taxa in the soil, and it is clearly unwise for host plants to secrete too much root exudate. We speculate that changes in the microbial community structure, involving a microecological imbalance in the rhizosphere soil, caused by the accumulation of ginseng root exudates is a key factor underlying ginseng soil sickness.

### 4.3. Root exudates, ginseng yield, ginseng quality, and fungal community interactions

Through the study of the plant-soil system we found that root exudates (amides, organic acids and esters) play a direct or indirect role in the growth and development of ginseng and ginsenoside synthesis. Little change in the ginseng rhizosphere fungal community structure occurred when the root exudate concentration in the soil was low; the increases in ginseng yield and quality in this situation may have been due to the direct involvement of root exudates in plant growth and metabolism rather than regulation of root-associated microorganisms (Dang et al., 2023). When the root exudate concentration in the soil exceeded a certain threshold (1.5 mg·g^−1^), the structure of the rhizosphere fungal community changed considerably; in this situation, the root exudates may have indirectly reduced the ginseng yield and quality by impacting the rhizosphere fungi. Similar roles played by root exudates have also been confirmed in other studies. For example, it has been shown that root exudates (oleamide, hexadecanamide, ethanolamine, etc.) act as a source of carbon and nitrogen, and their release and accumulation enhance competition between pathogens and other microorganisms and hinder healthy plant growth (Miao et al., 2023).

To further verify the relationships among ginseng yield, ginseng quality, and rhizosphere fungal communities under different root exudate concentrations, we conducted a correlation analysis (Figure 7). The results showed that soil fungal communities were closely related to ginseng yield and quality. Among them, the potential beneficial bacteria, represented by *Mortierella*, were significantly positively correlated with ginseng rhizome biomass and ginsenoside levels. Potential pathogens, represented by *Fusarium*, had strong negative correlations with rhizome biomass and ginsenoside levels. This suggests that the dominant fungi in the rhizosphere soil played an important role in ginseng growth and ginsenoside synthesis. The relationship between fungal communities and plant metabolism appears to involve a dynamic and complex biological process. Studies have shown that rhizosphere fungi can influence the accumulation of plant metabolites by regulating the expression of genes related to the immune system and the activity of enzymes involved in metabolic processes (Martínez-Castro et al., 2018; Begum et al., 2020). Overall, the results suggested that ginseng root exudates disrupt the distribution of rhizosphere soil fungi via stimulation/inhibition of specific rhizosphere fungal taxa. In particular, there was an increase in potentially pathogenic soil-borne fungi, reducing the ginseng yield and quality and representing an important cause of soil sickness. Finally, it should be noted that plant-soil microbe interactions should not be simply classified as generally beneficial or harmful, but rather according to whether they enhance plant adaptation to the environment under specific environmental conditions (Trivedi et al., 2020). For example, Zhou et al. (2023) found that intercropping systems (potatoonion and tomato) altered the composition of the tomato rhizosphere microbiome by promoting colonization by specific *Bacillus* species, and they subsequently isolated the *Bacillus* and demonstrated that it could inhibit the growth of the fungus *Verticillium dahliae* and enhance systemic resistance in tomato plants. Furthermore, Jin et al. (2023) demonstrated through transplantation experiments involving representative rhizosphere microbiota strains (*Sphingomonas* sp. S21, *Lysobacter* sp. L08 and *Pseudomonas* sp. P13) that they contributed to cadmium toxicity mitigation by biochar amendments and to the growth of tomato plants. Therefore, recognizing the limitations of this study, we will validate the results by transplanting representative microbial strains in future studies to clarify the specific microbial functions.

## 5. Conclusion

The interactions between plants and soil microorganisms involving root exudates involves an extremely complex series of processes. Recognizing this complexity, this study explored the effects of different root exudate concentrations on ginseng growth and development, ginsenoside synthesis, and rhizosphere fungal community structure, aiming to reveal the feedback behaviors that root exudates trigger in the plant-soil system. We found that low root exudate concentrations in the soil did not alter the rhizosphere fungal community structure, but had a beneficial effect on ginseng growth and ginsenoside synthesis. High root exudate concentrations significantly reduced ginseng yield and quality and led to changes in the soil fungal community structure and microecological imbalances, including an increase in the relative abundance of potentially pathogenic fungi. Overall, we speculate that continuous cropping of ginseng will lead to the accumulation of root exudates in the soil. When the concentration reaches a certain threshold, the allelopathic autotoxicity effect will cause plant-soil negative feedback-related outcomes such as soil microbial structure deterioration, disease aggravation, and nutrient imbalance, which may be the main reasons for the decrease in ginseng yield and quality. Therefore, we believe that by exploring new technologies and adopting a series of environmentally friendly green measures, such as planting structure adjustment and fallowing of arable land, we can manage soil sickness at source. Furthermore, if necessary, reducing the root exudate concentration in the soil, killing pathogenic bacteria, and replenishing beneficial bacteria may improve the yield and quality of ginseng and reduce soil sickness.

## Data availability statement

The datasets presented in this study can be found in online repositories. The names of the repository/repositories and accession number(s) can be found in the article/Supplementary material.

## Author contributions

JS: data curation, formal analysis, software, and writing—original draft. JY: methodology. SZ and QY: sample collection. LW: conceptualization, funding acquisition, and supervision. CX: conceptualization, project administration, funding acquisition, and writing—review and editing. All authors contributed to the article and approved the submitted version.

## Funding

This work was supported by the National Nature Science Foundation of China [81803649] and Science and Technology Project of Jilin Provincial Department of Science and Technology [20220204062YY].

## Conflict of interest

The authors declare that the research was conducted in the absence of any commercial or financial relationships that could be construed as a potential conflict of interest.

## Publisher’s note

All claims expressed in this article are solely those of the authors and do not necessarily represent those of their affiliated organizations, or those of the publisher, the editors and the reviewers. Any product that may be evaluated in this article, or claim that may be made by its manufacturer, is not guaranteed or endorsed by the publisher.
