# Supplementary material for: Root exudates influence rhizosphere fungi and thereby synergistically regulate Panax ginseng yield and quality
Source: Front Microbiol. 2023 Jul 20;14:1194224. doi: 10.3389/fmicb.2023.1194224 (PMC10397396; doi:10.3389/fmicb.2023.1194224)
Supplement: Supplementary file 1 [file Data_Sheet_1.pdf]

Supplementary Material

1 Supplementary Figures and Tables

1.1 Supplementary Figures

Supplementary Figure 1

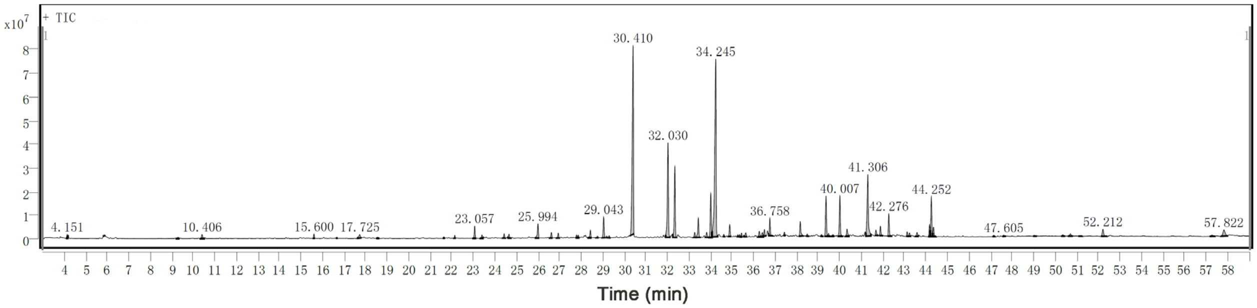

Figure S1. GC-MS chromatogram of ginseng root exudates.

Supplementary Figure 2

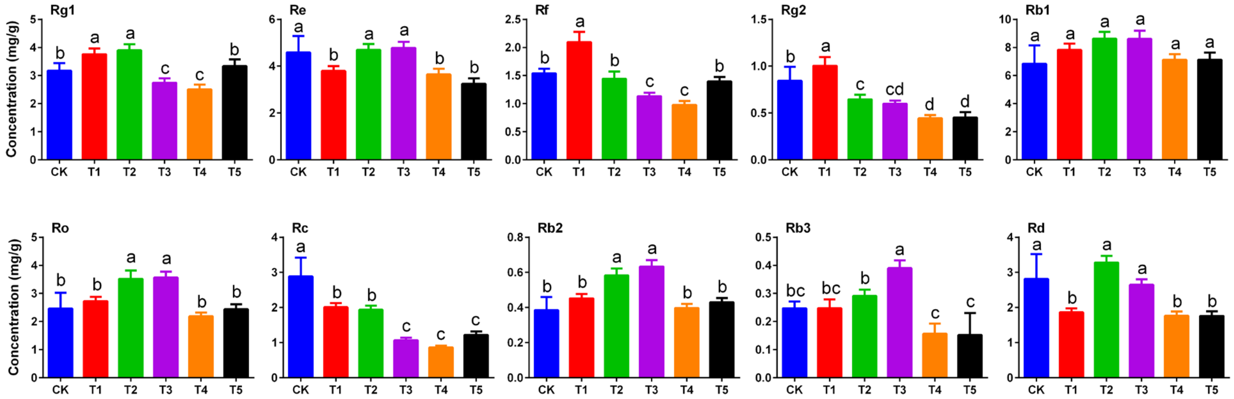

Figure S2. Effects of root exudate concentrations on ginsenoside concentrations in ginseng rhizomes (n = 3). Different letters indicate significant differences between treatments, $p < 0.05$. Root exudate concentrations: CK, 0 mg·g^{-1}; T1, 0.3 mg·g^{-1}; T2, 1.5 mg·g^{-1}; T3, 3 mg·g^{-1}; T4, 6 mg·g^{-1}; T5, 15 mg·g^{-1}.

Supplementary Figure 3

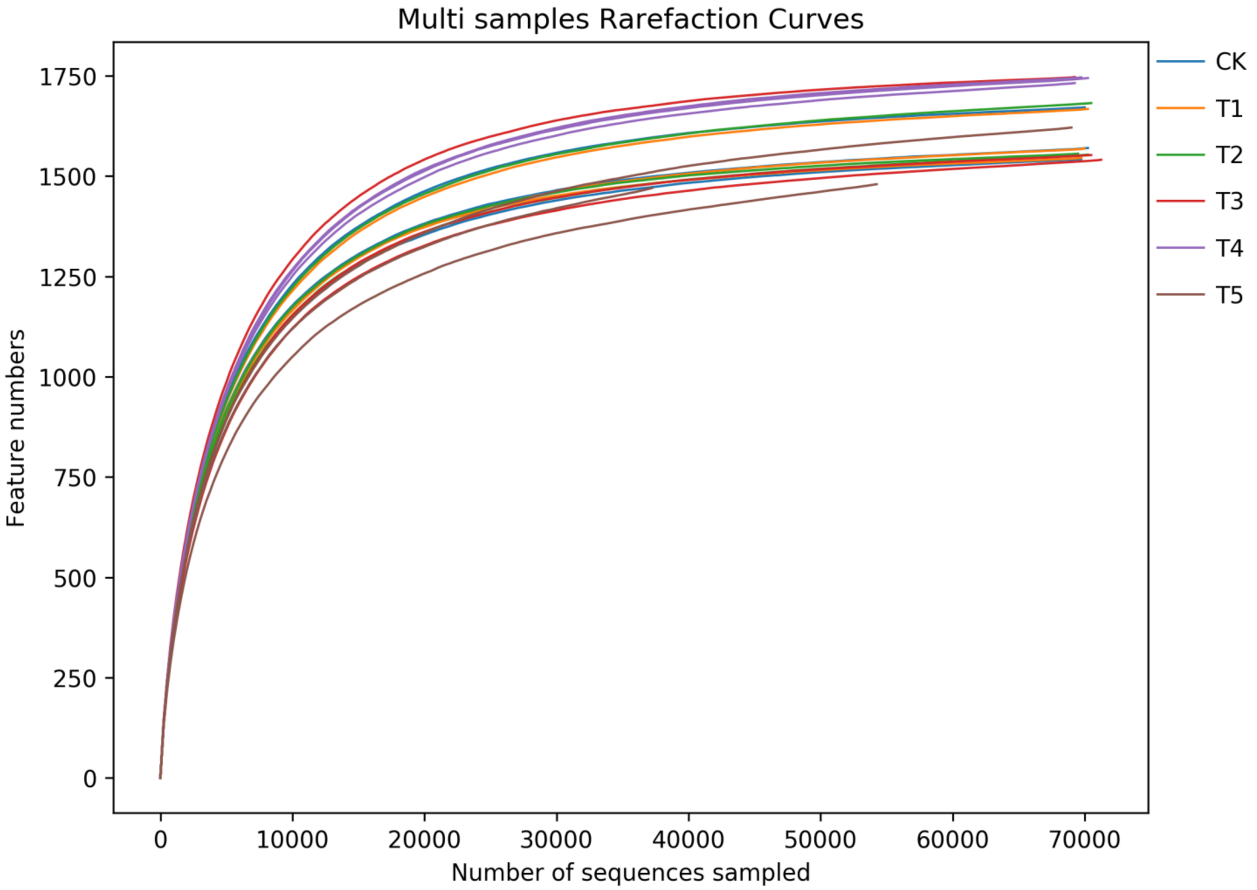

Figure S3. Rarefaction curves for all of the soil samples. Note: (a) Root exudate concentrations: CK, 0 mg·g^{-1}; T1, 0.3 mg·g^{-1}; T2, 1.5 mg·g^{-1}; T3, 3 mg·g^{-1}; T4, 6 mg·g^{-1}; T5, 15 mg·g^{-1}.

Supplementary Figure 4

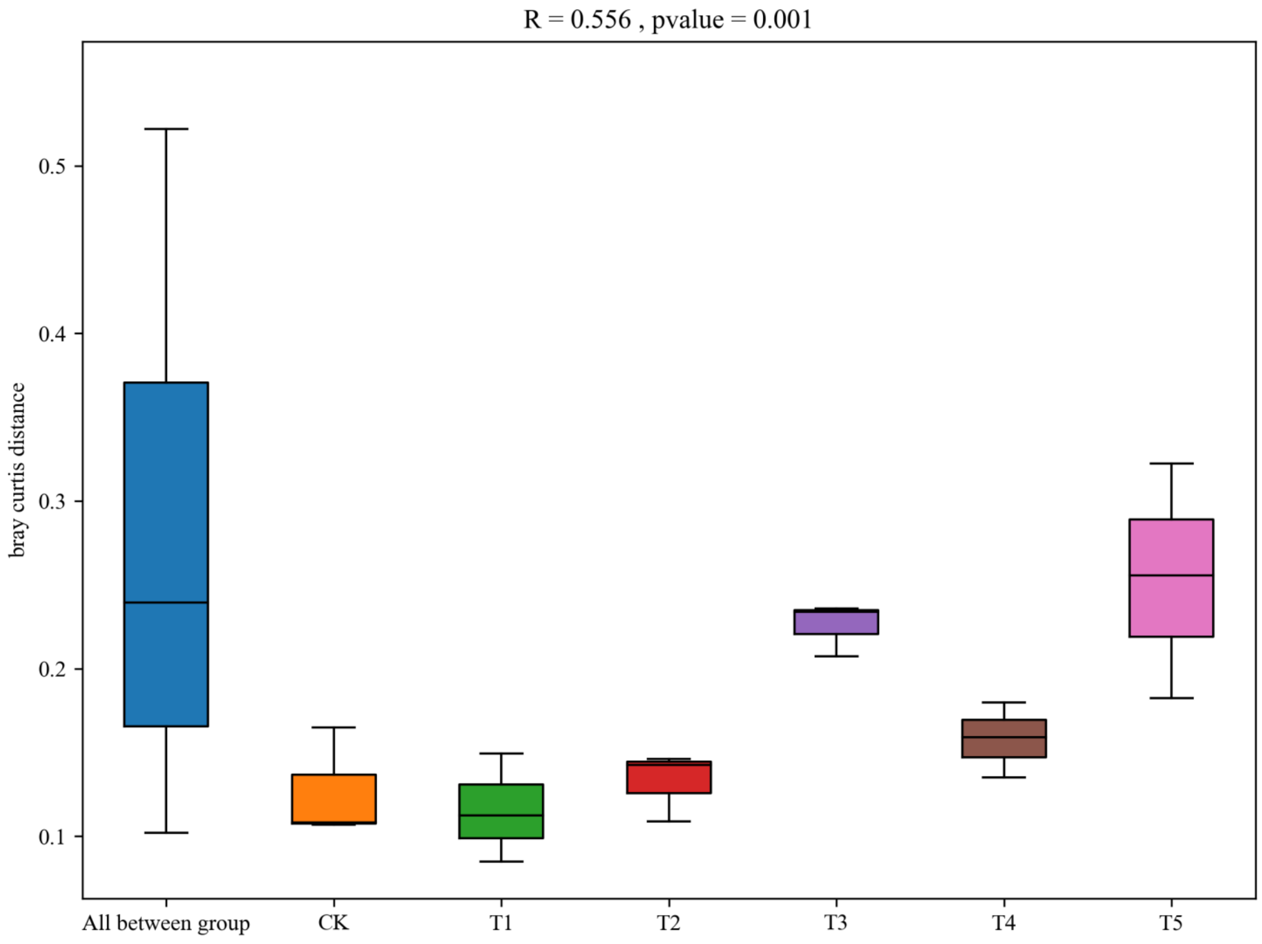

Figure S4. ANOSIM distance box plot. Note: (a) Root exudate concentrations: CK, 0 mg·g^{-1}; T1, 0.3 mg·g^{-1}; T2, 1.5 mg·g^{-1}; T3, 3 mg·g^{-1}; T4, 6 mg·g^{-1}; T5, 15 mg·g^{-1}.

1.2 Supplementary Tables

Supplementary Table 1

Table S1. Effects of root exudate concentrations on the proportion of each ginsenoside of all 10 ginsenosides in ginseng.

Treatment	Rg1%	Re%	Rf%	Rg2%	Rb1%	Ro%	Rc%	Rb2%	Rb3%	Rd%
CK	12	18	6	3	27	10	11	1	1	11
T1	15	15	8	4	30	11	8	2	1	7
T2	13	16	5	2	30	12	7	2	1	11
T3	10	18	4	2	33	14	4	2	1	10
T4	12	18	5	2	35	11	4	2	1	9
T5	15	15	6	2	33	11	6	2	1	8

Note: (a) Values are mean (n = 3). (b) Root exudate concentrations: CK, 0 $mg \cdot g^{-1}$; T1, 0.3 $mg \cdot g^{-1}$; T2, 1.5 $mg \cdot g^{-1}$; T3, 3 $mg \cdot g^{-1}$; T4, 6 $mg \cdot g^{-1}$; T5, 15 $mg \cdot g^{-1}$.

Supplementary Table 2

Table S2. Relative abundance of fungal phyla in the rhizosphere soil of ginseng under different root exudate concentrations.

genus	CK	T1	T2	T3	T4	T5
Ascomycota	62.63	62.14	62.43	64.53	67.27	74.04
Mortierellomycota	15.49	15.63	15.70	14.12	12.17	7.76
Basidiomycota	11.04	11.10	10.65	10.71	11.12	12.01
Glomeromycota	3.33	3.65	3.53	3.88	2.85	0.97
Chytridiomycota	1.43	1.41	1.41	1.16	1.28	0.85
Rozellomycota	0.53	0.58	0.52	0.61	0.71	0.91
Olpidiomycota	0.29	0.31	0.26	0.30	0.29	0.26
Mucoromycota	0.19	0.18	0.16	0.11	0.11	0.03
Zoopagomycota	0.06	0.06	0.04	0.05	0.06	0.09
Aphelidiomycota	0.01	0.04	0.02	0.01	0.01	0.00
Others	0.00	0.00	0.01	0.02	0.05	0.07
Unclassified	5.00	4.89	5.27	4.50	4.08	3.01

Note: (a) Values are mean (*n* = 3). (b) Root exudate concentrations: CK, 0 $mg \cdot g^{-1}$; T1, 0.3 $mg \cdot g^{-1}$; T2, 1.5 $mg \cdot g^{-1}$; T3, 3 $mg \cdot g^{-1}$; T4, 6 $mg \cdot g^{-1}$; T5, 15 $mg \cdot g^{-1}$.

Supplementary Table 3

Table S3. Relative abundance of genera in the rhizosphere soil of ginseng under different root exudate concentrations.

genus	CK	T1	T2	T3	T4	T5
Mortierella	14.62	14.75	14.81	13.17	11.36	7.20
Fusarium	5.78	5.44	5.83	6.53	6.82	8.26
Chaetomium	5.81	5.63	5.89	5.12	4.07	1.97
Aspergillus	2.94	2.98	2.93	3.51	4.11	5.67
Purpureocillium	3.50	3.66	3.69	3.24	2.51	0.90
Cladosporium	1.49	1.47	1.42	1.62	1.98	2.94
Metacordyceps	2.13	2.14	2.13	1.71	1.24	0.31
Penicillium	1.09	0.97	1.09	1.24	1.74	2.84
Stachybotrys	1.83	1.87	1.81	1.64	1.18	0.44
Monascus	1.20	1.36	1.28	1.43	1.60	1.88
Others	37.58	37.63	36.57	39.04	41.64	46.89
Unclassified	22.04	22.11	22.54	21.76	21.75	20.72

Note: (a) Values are mean (n = 3). (b) Root exudate concentrations: CK, 0 $mg\cdot g^{-1}$; T1, 0.3 $mg\cdot g^{-1}$; T2, 1.5 $mg\cdot g^{-1}$; T3, 3 $mg\cdot g^{-1}$; T4, 6 $mg\cdot g^{-1}$; T5, 15 $mg\cdot g^{-1}$.
